# Fully Automated Lumen Segmentation Method for Intracoronary Optical Coherence Tomography

**DOI:** 10.1155/2018/1414076

**Published:** 2018-12-26

**Authors:** Elżbieta Pociask, Krzysztof Piotr Malinowski, Magdalena Ślęzak, Joanna Jaworek-Korjakowska, Wojciech Wojakowski, Tomasz Roleder

**Affiliations:** ^1^Department of Biocybernetics and Biomedical Engineering, AGH University of Science and Technology, Al. Mickiewicza 30, 30-059 Krakow, Poland; ^2^Jagiellonian University Medical College, Faculty of Health Science, Institute of Public Health, Krakow, Poland; ^3^KCRI, Krakow, Poland; ^4^Department of Automatic Control and Robotics, AGH University of Science and Technology, Al. Mickiewicza 30, 30-059 Krakow, Poland; ^5^Third Department of Cardiology, Medical University of Silesia, Katowice, Poland

## Abstract

**Background:**

Optical coherence tomography (OCT) is an innovative imaging technique that generates high-resolution intracoronary images. In the last few years, the need for more precise analysis regarding coronary artery disease to achieve optimal treatment has made intravascular imaging an area of primary importance in interventional cardiology. One of the main challenges in OCT image analysis is the accurate detection of lumen which is significant for the further prognosis.

**Method:**

In this research, we present a new approach to the segmentation of lumen in OCT images. The proposed work is focused on designing an efficient automatic algorithm containing the following steps: preprocessing (artifacts removal: speckle noise, circular rings, and guide wire), conversion between polar and Cartesian coordinates, and segmentation algorithm.

**Results:**

The implemented method was tasted on 667 OCT frames. The lumen border was extracted with a high correlation compared to the ground truth: 0.97 ICC (0.97–0.98).

**Conclusions:**

Proposed algorithm allows for fully automated lumen segmentation on optical coherence tomography images. This tool may be applied to automated quantitative lumen analysis.

## 1. Introduction

Today's medical practice diagnosis of coronary artery disease (CAD) is made using mostly invasive imaging modalities among which coronary angiography is the most popular one, being currently considered the standard during cardiac catheterization and hemodynamic assessment. However, coronary angiography produces “luminogram” delineating only the shape of the contrast-filled lumen without any information about plaque morphology or vessel wall [1]. This is why most recently angiography is accompanied by newer intravascular imaging techniques like IVUS and OCT which employ acoustic waves and near-infrared light, respectively, in order to generate cross-sectional, volumetric images of coronary arteries [2]. OCT provides images of high contrast and very high spatial resolution (10–20 *µ*m), 10 times higher than IVUS, thus allowing characterization of atherosclerotic plaques and assessment of coronary stenting including stent apposition and struts coverage [3, 4]. One of the main challenges in OCT image analysis is the accurate detection of lumen which is significant for the further prognosis.

This paper is organized in 4 sections as follows: Section 1.1 presents the motivation of this work and the review of the state of the art in the area of lumen segmentation.  Section 2 specifies the overview of the implemented algorithm. The conducted statistical analysis, results, and discussion of the achieved outcomes are presented in Section 3. At last, Section 4 closes the paper and highlights future directions.

### 1.1. Motivation

OCT images clearly depict the boundaries between lumen and vessel, which facilitate image interpretation. Currently image processing has been mainly conducted manually by Core Lab analysts, but due to large number of cross sections in OCT image sequence, this is usually a time-consuming process with high inter-intraobserver variability [5]. However, above limitations can be addressed by introducing automatic image analysis including detection of lumen contours [6, 7]. Lumen segmentation is the first but crucial step in the image analysis process as it allows detection of stenosis and high-risk plaques. It has been addressed not only for OCT pullbacks but also for IVUS image sequences [8, 9].

### 1.2. Related Works

Automatic lumen contour detection can be a very challenging step as OCT images typically contain various artifacts like guide wire shadowing, motion artifacts, bifurcations, or nondiluted intraluminal blood. As the analysis of OCT images is a demanding process, many automatic methods have been developed for lumen detection in OCT [2, 10–16] in recent years.

These methods usually employ multistep image processing techniques including binarization approach [10, 11, 16], morphological operations [10, 11], intensity curve methods [16], Markov random field (MRF) model [2], or wavelet transform [12].

Different OCT technologies, various image textures, diffused and complex lesions and, furthermore, not well diluted blood from vessels have a huge impact on segmentation outcome as well as feature extraction in above described methods [2, 8–10]. Additionally, images with poor luminal or substantial luminal blood in contact with the arterial wall cannot be well delineated by active contour methods [14].

Methods developed and proposed by other research teams tend to be very accurate and have good computational cost. But, they applied them for high-quality images including only one kind of individual artifacts. Due to these obstacles, there is still room for a complex solution which could improve the segmentation process for most cases.

Diffused and complex lesions have motivated the development of the proposed method for OCT analyses with a new sequence of morphological operations, and interpolation methods which have been designed to reconstruct lumen object, resulting in a more accurate segmentation outcome, even in the presence of bifurcation structures and not well-diluted intraluminal blood. Most of the listed above methods can only be applied on the healthy or nonbifurcation images [11] or for good quality images without artifacts [10, 16].

Manual segmentation by independent observers is mainly used as the reference for particular method validation. To increase the value of our work, we have compared our outcomes with two widely used, commercially available systems (Medis medical imaging systems and St. Jude Medical OPTIS integrated system). Moreover, the test was conducted on the same dataset for the results to be reliable. This algorithm achieved higher classification results compared to existing OCT segmentation programs, scoring 0.97 ICC in lumen area compared with a gold standard ground-truth method.

In this paper, we propose a fully automated method to segment the lumen area in run OCT pullbacks without excluding any frames. Our solution can be used to analyze poor quality images as well as images with diseased vessels and bifurcations.

## 2. Materials and Methods

**Figure 1:**
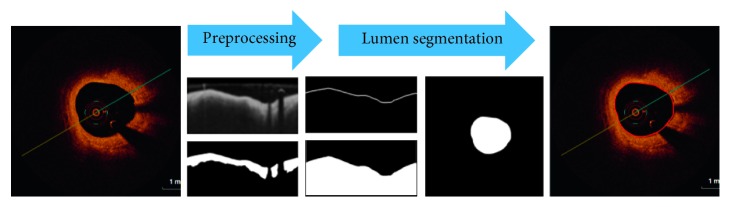
Proposed methodology for automatic lumen contour detection on OCT images. The flowchart shows the major steps of the detection process including preprocessing and segmentation with their outcomes.

The proposed automated lumen detection algorithm on intracoronary optical coherence tomography images consists of two main stages: preprocessing (image enhancement and artifacts removal) and lumen segmentation with contour correction. Image analysis has been implemented in Matlab software using the Image Processing Toolbox, where the flowchart of the proposed algorithm is presented in Figure 1.

### 2.1. Database Specification

The analyzed material is composed of 667 frames of different patients, from the Medical University of Silesia. Images used in this study were acquired by the FD-OCT system (C7-XR system OCT Intravascular Imaging System, Westford, MA) and two kinds of imaging catheters: the C7 Dragonfly and Dragonfly OPTIS catheter with automatic pullback, drive motor optical controller. The analyzed data were obtained with the pullback speed of 20 mm/s and 18 mm/s, respectively.

**Figure 2:**
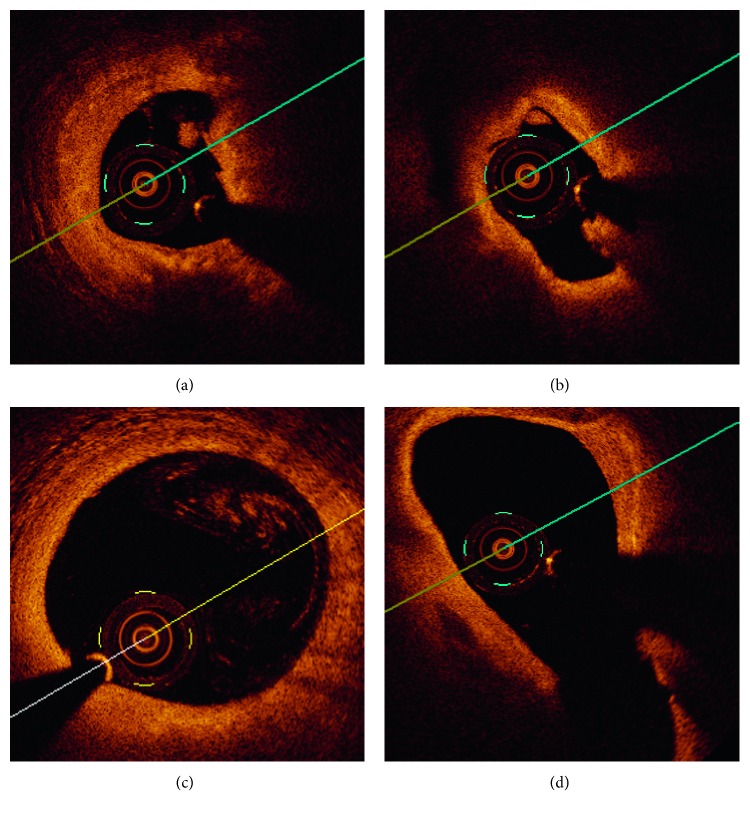
Examples of OCT cross-sectional view: (a), (b) lumen irregularities with visible thrombus, (c) residual blood, and (d) bifurcations.

The chosen images contain a variety of vessel features like lumen irregularities caused by intraluminal masses (thrombus), branches, or different intensity profiles due to not well-diluted blood (Figure 2).

### 2.2. OCT Image Preprocessing

**Figure 3:**
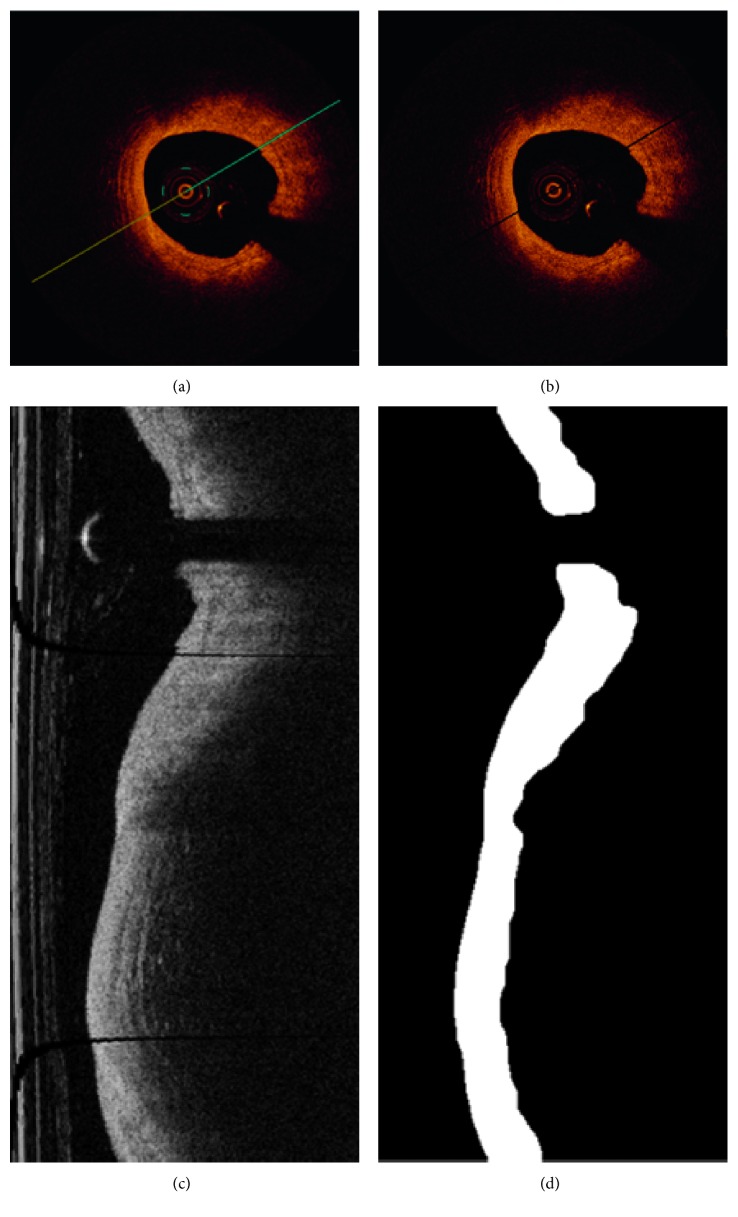
Output of the preprocessing step: (a) OCT input image, (b) artifact removal, (c) OCT image after polar transformation, and (d) primary segmentation of the lesion in polar coordinates.

OCT images are inhomogeneous, complex (variation in degree of intensity and shape), and furthermore they contain extraneous artifacts, such as bright concentric circular rings and bright structure from guide wire with a characteristic shadow behind it. These types of artifacts appear in almost every frame which makes the advanced image analysis steps impossible. Therefore, the preprocessing stage is necessary to obtain the binary image of the intimal layer (the most inner layer of three layers building the vessel wall) without artifacts from the diagnostic catheter and improve the quality of the image for further analysis (Figure 3).

The proposed algorithm receives as an input each frame in turn from the whole OCT image dataset. Multiframe images have been saved in DICOM format (pullback run), and each frame is a 2D RGB image in a Cartesian coordinate system.

Firstly, all calibration markers and text remarks are removed from the image using a mask of the pixels that are colored. The analyzed RGB image is converted into grayscale with the NTSC 1953 standard, which converts RGB values to grayscale values by forming a weighted sum of the R, G, and B components.

After converting the RGB image to the grayscale image, the polar transform is applied, and further preprocessing stages are being performed in polar coordinates. This transformation allows us to convert the circular shape of the coronary artery visible in a cross-sectional view to a straightened structure. In mathematics, the polar coordinate system is a two-dimensional coordinate system in which each point on a plane is determined by a distance from a reference point and an angle from a reference direction.

The ring shape distortion from the imaging catheter in polar space is shown as the straightened structure on the left side of the image with the known size—Dragonfly catheter with a diameter of 2.7 French which gives 0.91 mm. Using the knowledge about spatial resolution of the image and catheter diameter, we can calculate the region of structure and remove it from the image. Another significant artifact, which may limit the segmentation process, is a speckle noise from not well-diluted blood. Speckle noise may affect the lumen segmentation outcome by classifying it mistakenly as a tissue resulting in underestimated real lumen area.

In order to remove any destructive speckle effects without damaging borders, we use a median filter with a 5 × 5 window [17]. After median filtering, the Gaussian smoothing operator is used to “blur” the image, aiming at removing unnecessary details and reducing noise from background. The Gaussian smoothing operator is a 2-D convolution operator that uses a kernel of Gaussian values representing the shape of a Gaussian (bell-shaped) hump.

The Gaussian filter is a low-pass filter, attenuating high-frequency signals. It calculates a weighted average of each pixel's neighborhood, with the average weighted more towards the value of the central pixels, and a Gaussian distribution provides gentler smoothing and preserves the better edges [18].

An automatic thresholding on polar space is used to generate a new binary image with clearly separated region with high-gradient magnitude-intimal layer.

### 2.3. Lumen Segmentation and Contour Drawing

Methods and algorithms developed for segmentation of medical structures are specific to application, imaging modality, and type of body part to be studied. Because of image complexity, there is no perfect method to segment all of the medical structures with high efficiency. However, the success of the lumen segmentation step is crucial for the further analysis of OCT images and correct diagnosis.

**Figure 4:**
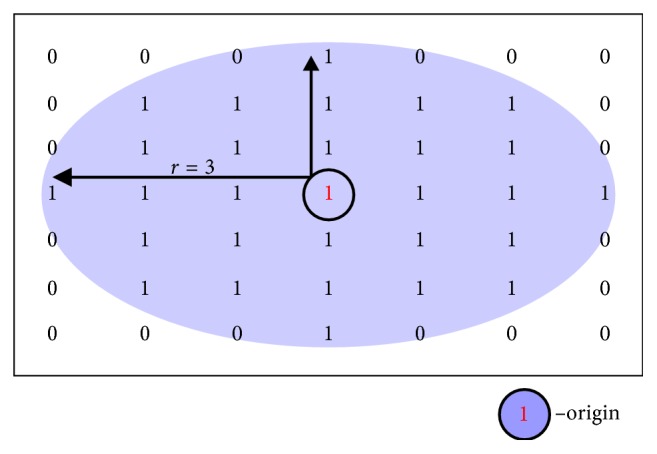
Illustration of a flat structuring element [20].

The outcome of the preprocessing stage is a binary image with the primary segmented area that still contains small artifacts like insufficiently diluted blood close to the imaging catheter. To minimize the effect of artifacts on the final result, we subject the image to morphological opening and closing operations. While erosion and dilation have the major disadvantage of changing the size of our region of interest, opening and closing retain the interesting area. Opening and closing are basic methods of morphological noise removal. Opening removes small, unwanted objects from the foreground placing them in the background, while closing fills small holes and connects disjoint objects in the foreground, changing small areas of background into foreground [19]. Based on the lumen shape, we use a disk-shaped structuring element to preserve the circular nature of the object. The disk-shape element is a flat, structuring element, where *R* specifies the radius (Figure 4).

**Figure 5:**
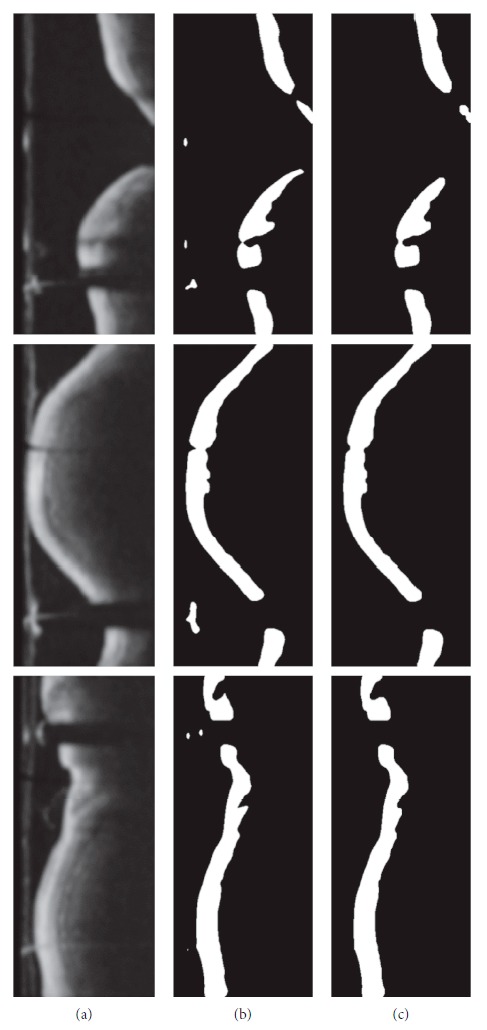
Examples of the results after morphological opening and closing operations on OCT images in polar coordinates: (a) images after Gaussian filtering, (b) images after binarization, and (c) images after morphological opening and closing operations.

The radius was determined experimentally and set to 5.  Figure 5 shows examples of the results of morphological opening and closing operations.

**Figure 6:**
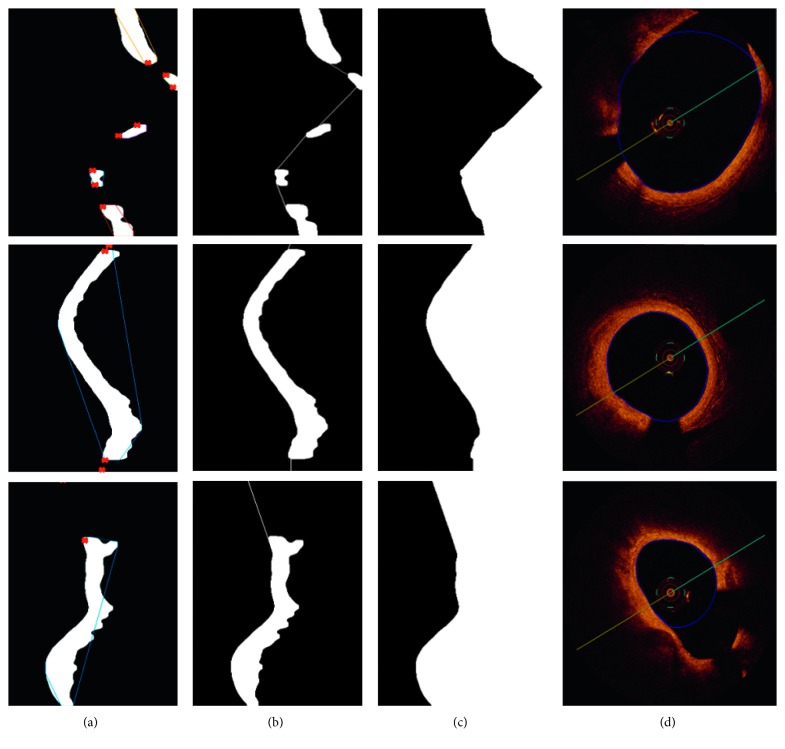
Examples of chosen extrema points needed to perform linear interpolation: (a) binary image after preprocessing and artifact removal with marked extrema, (b) extrema point connection (linear interpolation), (c) lumen segmentation outcome, and (d) input OCT image with lumen contour tracing. Images (a)–(c) are in polar coordinates, and image (d) is after transformation to Cartesian coordinates.

Artifacts from the imaging catheter and the guide wire were removed in the preprocessing stage. However, the shadow from guide wire makes the intimal layer discontinuities what can be observed in Figure 3(c). A similar effect is caused by bifurcations. The gap from guide wire shadow is usually of the same size, regular, and easily to be found and filled. More problematic are gaps caused by bifurcations which can vary in size, and additionally, the remained objects of the segmented lumen may have irregular shapes. Bifurcation results in lumen area distortion can be observed in Figure 6(d). The interpolation of remaining regions is necessary to draw the final lumen contour which should be as close as possible to the expected values. To solve this problem, we have applied a modified version of linear interpolation which is tailored to our needs.

**Figure 7:**
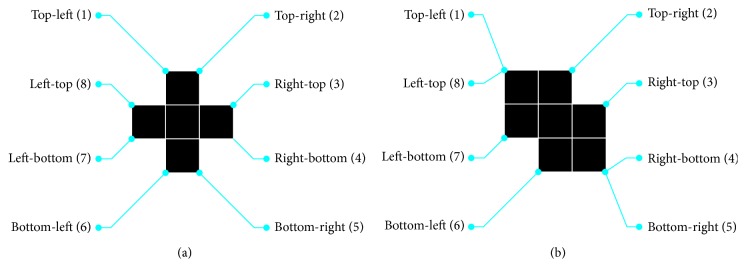
Illustration of marked extremes for two different regions [20].

In order to connect the parts of lumen, we analyze the boundary information (location and coordinates) of every disconnected part of intima layer (traces of the exterior boundaries of the object). We receive a cell array of boundary pixel coordinates of all the objects in the binary image [20]. To perform the linear interpolation, extreme points are calculated as presented in Figure 7.

Few of the extrema points are candidates to the contour points including bottom-left, top-left, and top-right. We analyze the objects from the top to the bottom. Following points are being interpolated: for the first, upper object, we select the left-bottom point, and for the second object which is located below, we choose between the top-left and top-right points. The final choice is determined by the value of Euclidean distance between extremes. The bigger the bifurcation is, the longer the distance will be. The individual extreme coordinates are taken to calculate the distance and perform linear interpolation. We experimentally checked that the cutoff point for bifurcation is 2 mm size.  Figure 6 presents some examples of chosen contour points. To avoid sharp contour reconstructions, additional points have been chosen by moving up and down from extreme points and finding the first white pixel in the current row.

**Figure 8:**
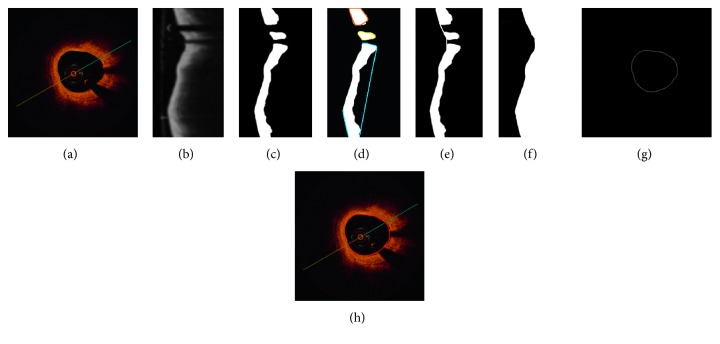
Lumen segmentation steps: (a) original image in Cartesian coordinates; (b) image after transformation to polar coordinates and after catheter removal applying Gaussian filtering; (c) image in polar coordinates after binarization and morphological operations, small artifacts are removed and small gaps filled; (d) Image in polar coordinates with marked extrema; (e) based on extrema, connection points are chosen and linear interpolation is applied to fill all gaps; (f) lumen segmentation outcomes; (g) segmented contour transformed back to Cartesian coordinates and after smoothing filter; (h) final image, cross-sectional view with marked contour.

After setting the contour points, a linear interpolation is used. In that way, all discontinuities (bifurcations, shadow from guide wire and from artifacts) are filled. Throughout this method, the lumen border line in the polar image was obtained (Figure 6(c)). Finally, the lumen border points are detected by the Sobel edge detection algorithm [21]. The Sobel operator performs a 2-D spatial gradient measurement and emphasizes regions of high spatial frequency that corresponds to edges. After all operations have been carried out, the resulting polar image is transformed into an image in Cartesian coordinates.  Figure 8 shows each step of lumen segmentation.

As the segmentation outcome, the resulting contour does not have the smoothness that the vessel is expected to have. The Savitzky–Golay sliding polynomial filter with window width 35 and polynomial order 2 [22] is being applied.

Savitzky and Golay showed that a set of integers (*A*_−*n*_, *A*_−(*n* − 1)_,…, *A*_(*n* − 1)_, *A*_*n*_) could be derived and used as weighting coefficients to carry out the smoothing operation [23]. The use of these weighting coefficients, known as convolution integers, is exactly equivalent to fit the data to a polynomial. Therefore, the smoothed data point (*y*_*k*_)_s _ by the Savitzky–Golay algorithm is given by the following equation:

(1) $$\begin{array}{c} {\left(y_{k}\right)}_{\text{s }}=\frac{\sum_{i=-n}^{n}A_{i}y_{k+1}}{\sum_{i=-n}^{n}A_{i}}, \end{array}$$

where *A*_*i*_ are weighting coefficients to perform the smoothing operation.

## 3. Results

**Figure 9:**
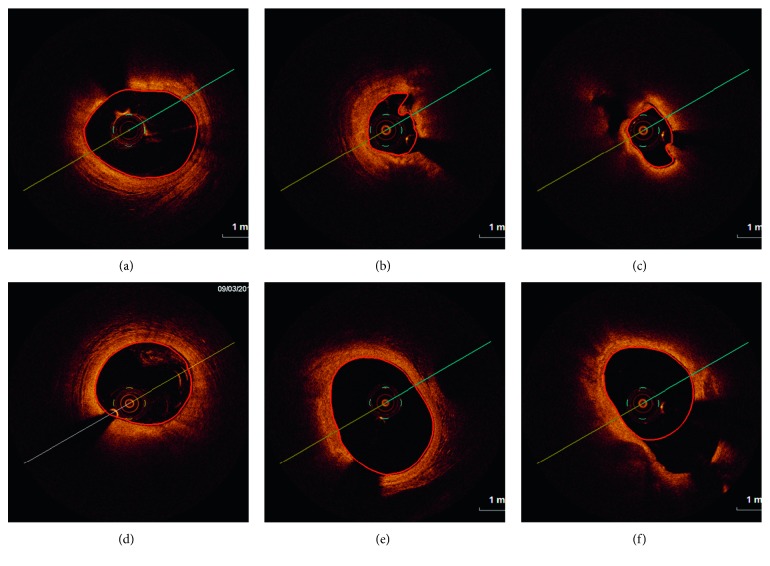
Results of the described lumen segmentation algorithm dedicated for OCT images. Presented images show six different cases including various artifacts and difficulties.

The validation of the described fully automated lumen segmentation method has been performed on 667 intravascular optical coherence tomography frames from different patients. The data were provided by the Medical University of Silesia.  Figure 9 presents the achieved results.

### 3.1. Statistical Analysis

Statistical analysis involves data obtained from four methods: our algorithm, two commercially available systems, and manual analysis (ground truth mask). Continuous parameters were reported as mean and median with the first and the third quartiles (Q1: 25%; Q3: 75%).

The Bland–Altman analysis was used to assess the agreement between two measurement methods. It is a method comparison technique proposed by Altman and Bland [24] based on the quantification of the agreement between two quantitative measurements by studying the mean difference and constructing limits of agreement.

The results for the particular measurements were presented as mean with 95% confidence interval and as median with the first and the third quartiles. Discrepancies between the first and the second analysis were calculated as absolute and relative differences and presented as means with 95% CIs. Intraclass correlations were calculated as the main measure of agreement along with the graphical representation as the Bland–Altman plots. Analyses for statistical computing were performed in R language (R Core Team 2017, Vienna, Austria).

### 3.2. Validation of Automated Lumen Segmentation

In order to validate the described algorithm, we compare four lumen detection methods: our solution, ground truth mask, and two commercially available systems including St. Jude Medical and System (system 1) and Medis medical imaging systems (system 2). Manual segmentation has been performed by independent observers-interventional cardiologists with extensive clinical experience. Furthermore, our experts were involved in the development of methodology and results analysis.

The following parameters have been analyzed for each of the described methods: lumen area, mean lumen diameter, minimal lumen diameter, and maximal lumen diameter, and are collected in Table 1.

The results of the assessed parameters are collected in Tables 2–4 and presented by the Bland–Altman plots.

To enable the analysis of statistical data, the following parameters have been collected additionally.

The relative difference is calculated using the following equation:

(2) $$\begin{array}{c} \text{RD}=\frac{\sum_{i=1}^{N}\left(I_{i}-\;\;O_{i}/\max\left(O_{i},\;\;I_{i}\right)\right)}{N}\;\;*100\%. \end{array}$$

The absolute relative difference is calculated using

(3) $$\begin{array}{c} \text{ARD}=\;\;\frac{\sum_{i=1}^{N}\left(\left|I_{i}-\;\;O_{i}\right|/\max\left(O_{i},\;\;I_{i}\right)\right)}{N}*100\%, \end{array}$$

where *N* is the total number of frames, *i* is the number of current frame, *O_i_* is the value for 1st measurement, and *I*_*i*_ is the value for 2nd measurement.

### 3.3. Discussion of the Results

The lumen detection was performed on a desktop computer with an Intel Core i5-4200, 1.60 GHz processor, 8 GB RAM, Windows 10 64 bits, and Matlab (R2016b). The average time of the lumen contour detection was 1.099 s. The average time of manual segmentation of a slice was approximately 60 seconds. As it is shown, the computer-aided segmentation systems is much faster than the manual segmentation; furthermore, it is objective to the same cases and also very accurate. To validate our proposed method, we tested the same dataset with results from manual analyses and two commercially available tools for automatic lumen detection. We achieved high correlation in lumen area compared with a gold standard ground-truth method (manual analyses performed by an expert): 0.97 ICC. The results in the literature [2, 11, 12] reported an absolute difference of the mean lumen area of 0.1 mm^2^. De Macedo et al. [11] obtained absolute difference of mean lumen area of 0.17 mm^2^. Our proposed method showed similar results (absolute difference of mean lumen area of 0.1 mm^2^) to those presented previously published methods, but what is worth to highlight in our validation process is that all frames were included to analyse even frames containing complex plaque, artifacts from residual blood, or bifurcations with diameters > 2 mm. Furthermore, as we can see the parameters calculated by our methods are similar to obtained results from both commercially available systems (system 1 and system 2), the systems have not been described in any paper. Our algorithm can be easily implemented again and tested on a new dataset.

**Figure 10:**
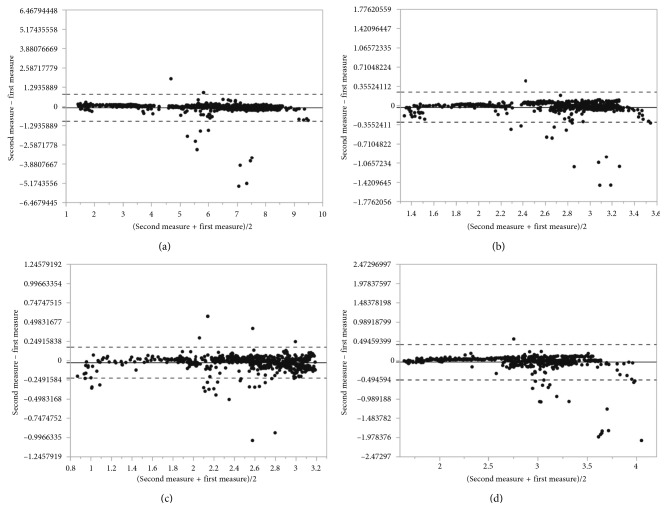
Bland–Altman plot for (a) lumen area, (b) mean lumen diameter, (c) minimal lumen diameter, and (d) maximal lumen diameter between our method and ground truth method.

**Figure 11:**
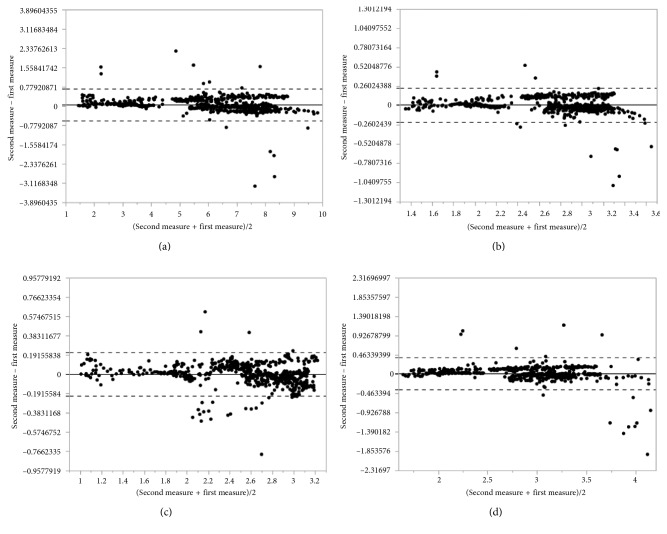
Bland–Altman plot for (a) lumen area, (b) mean lumen diameter, (c) minimal lumen diameter, and (d) maximal lumen diameter between our method and automated lumen detection proposed by commercially available system 1.

**Figure 12:**
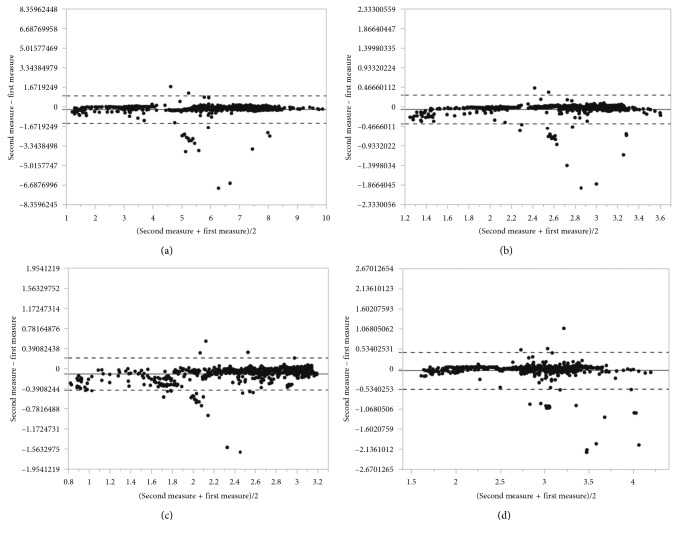
Bland–Altman plot for (a) lumen area, (b) mean lumen diameter, (c) minimal lumen diameter, and (d) maximal lumen diameter between our method and automated lumen detection proposed by commercially available system 2.

The Bland–Altman plots (Figures 10–12) indicate a good agreement between used methods, where the solid line denotes the mean difference between the first and the second measurement, while the dashed lines indicate ±1.96 standard deviation. Most points plotted are between the solid line (mean diff) and the dashed line (mean ± 2 ∗ standard deviation).

An absolute difference of mean lumen area calculated between our method and automated lumen detection proposed by system 1 is of 0.06 mm^2^ compared with system 2 results of absolute difference of mean lumen area of 0.22. Although the lumen areas are similar in all methods and there is high ICC between our method and the others (0.95–0.99), the lumen diameters are shown with lower ICC, especially between our method and system 2.

In terms of limitations, our method was not tested on images with the presence of stents which could have a negative impact on our algorithm. This limitation may be solved in the future by developing methods to extract the struts and fill the artifacts from strut shadows.

## 4. Conclusion

We presented a fully automated methodology which is able to detect and draw correctly lumen contours in OCT images including frames with bifurcations and artifacts from blood. The automated method was validated using the manual analyzes performed by an Expert as a gold standard as well as commercially available tools. The results suggest that our method can be a useful tool for vessel segmentation and further analysis. The achieved results indicate that the proposed algorithm fulfills the requirement.

## Figures and Tables

**Table 1:** Calculated parameters for each of the analyzed methods.

	Our method mean	System 1 mean	System 2 mean	Ground truth mean
Lumen area (mm^2^)	5.99	6.04	5.76	5.89
Mean lumen diameter (mm)	2.72	2.72	2.66	2.68
Minimal lumen diameter (mm)	2.52	2.52	2.42	2.49
Maximal lumen diameter (mm)	2.91	2.93	2.87	2.88

**Table 2:** Statistical comparison of the parameters between our methodology and manual analyses by analyst (ground truth).

	Our method (first measure) mean (CI)	Ground truth (second measure) mean (CI)	Our method (first measure) median (IQR)	Ground truth (second measure) median (IQR)	Difference	Relative difference	ICC (95% CI)	ICC *p* value
Lumen area	5.99 (5.83–6.14)	5.89 (5.74–6.04)	6.45 (4.72–7.67)	6.29 (4.67–7.50)	0.10 (0.06–0.13)	−1.12% (−1.55% to −0.68%)	0.97 (0.97–0.98)	<0.0001
Mean lumen diameter	2.72 (2.68–2.76)	2.68 (2.64–2.72)	2.88 (2.41–3.13)	2.82 (2.43–3.09)	0.03 (0.02–0.04)	−1.15% (−1.48% to −0.83%)	0.96 (0.95–0.97)	<0.0001
Minimal lumen diameter	2.52 (2.48–2.56)	2.49 (2.45–2.53)	2.64 (2.18–2.96)	2.61 (2.10–2.93)	0.03 (0.02–0.03)	−1.11% (−1.44% to −0.78%)	0.98 (0.98–0.98)	<0.0001
Maximal lumen diameter	2.91 (2.87–2.96)	2.88 (2.84–2.92)	3.07 (2.54–3.30)	3.01 (2.58–3.25)	0.04 (0.02–0.05)	−0.81% (−1.23% to −0.39%)	0.91 (0.89–0.92)	<0.0001

**Table 3:** Statistical comparison of parameters between our methodology and commercially available system 1.

	Our method (first measure) mean (CI)	System 1 (second measure) mean (CI)	Our method (first measure) median (IQR)	System 1 (second measure) median (IQR)	Difference	Relative difference	ICC (95% CI)	ICC *p* value
Lumen area	5.99 (5.83–6.14)	6.04 (5.90–6.19)	6.45 (4.72–7.67)	6.50 (5.01–7.56)	−0.06 (−0.08–−0.03)	1.67% (1.25%–2.10%)	0.99 (0.98–0.99)	<0.0001
Mean lumen diameter	2.72 (2.68–2.76)	2.72 (2.68–2.76)	2.88 (2.41–3.13)	2.87 (2.52–3.10)	−0.00 (−0.01–0.00)	0.34% (0.06%–0.63%)	0.98 (0.97–0.98)	<0.0001
Minimal lumen diameter	2.52 (2.48–2.56)	2.52 (2.48–2.56)	2.64 (2.18–2.96)	2.52 (2.48–2.56)	0.00 (−0.01–0.01)	0.16% (−0.16%–0.49%)	0.98 (0.97–0.98)	<0.0001
Maximal lumen diameter	2.91 (2.87–2.96)	2.93 (2.89–2.97)	3.07 (2.54–3.30)	3.08 (2.66–3.28)	−0.02 (−0.03–−0.00)	0.82% (0.43%–1.21%)	0.94 (0.93–0.95)	<0.0001

**Table 4:** Statistical comparison of parameters between our methodology and another commercially available system 2.

	Our method (first measure) mean (CI)	System 2 (second measure) mean (CI)	Our method (first measure) median (IQR)	System 2 (second measure) median (IQR)	Difference	Relative difference	ICC (95% CI)	ICC *p* value
Lumen area	5.99 (5.83–6.14)	5.76 (5.61–5.92)	6.45 (4.72–7.67)	6.13 (3.98–7.48)	0.22 (0.18–0.27)	−4.23% (−4.88% to −3.57%)	0.95 (0.93–0.97)	<0.0001
Mean lumen diameter	2.72 (2.68–2.76)	2.66 (2.61–2.70)	2.88 (2.41–3.13)	2.79 (2.25–3.09)	0.06 (0.05–0.07)	−2.42% (−2.85% to −1.99%)	0.94 (0.92–0.96)	<0.0001
Minimal lumen diameter	2.52 (2.48–2.56)	2.42 (2.37–2.46)	2.64 (2.18–2.96)	2.56 (2.01–2.91)	0.10 (0.09–0.12)	−4.96% (−5.53% to −4.39%)	0.95 (0.85–0.97)	<0.0001
Maximal lumen diameter	2.91 (2.87–2.96)	2.87 (2.83–2.91)	3.07 (2.54–3.30)	3.00 (2.50–3.26)	0.04 (0.02–0.06)	−1.11% (−1.58% to −0.63%)	0.89 (0.88–0.91)	<0.0001

## Data Availability

No data were used to support this study.
